# Metformin-Associated Lactic Acidosis Induced by Even Modest Amounts of Alcohol: A Case Report

**DOI:** 10.7759/cureus.95194

**Published:** 2025-10-22

**Authors:** Yuichi Miyata, Kiyomitsu Fukaguchi, Hiroshi Koyama, Masatoshi Nakamura

**Affiliations:** 1 Department of Pharmacy, Shonan Kamakura General Hospital, Kamakura, JPN; 2 Department of Critical Care, Shonan Kamakura General Hospital, Kamakura, JPN; 3 Department of Emergency Medicine, Shonan Kamakura General Hospital, Kamakura, JPN

**Keywords:** alcohol, case report, diabetes mellitus type 2, lactic acidosis, metformin

## Abstract

A 66-year-old man with type 2 diabetes mellitus, treated with metformin, experienced loss of consciousness after bathing and consuming approximately 700 mL of beer (estimated 28 g of alcohol). He had no history of habitual heavy drinking. Upon arrival at the emergency department, he was alert (Glasgow Coma Scale score E4V5M6) with a heart rate of 73 bpm (sinus rhythm), blood pressure of 68/43 mmHg, respiratory rate of 18 breaths per minute, and oxygen saturation of 98% on room air. Arterial blood gas analysis revealed a pH of 7.33, a bicarbonate level of 16.2 mmol/L, and a lactate level of 7.4 mmol/L, consistent with lactic acidosis. These findings indicate a significant metabolic disturbance despite only modest alcohol intake. Metformin-associated lactic acidosis (MALA) was suspected, and he was admitted to the intensive care unit. Despite fluid resuscitation, hypotension required a norepinephrine infusion, which was discontinued after 12 hours. The acidemia improved, and all medications except metformin were resumed. He was discharged on hospital day 3 with stable renal function. This case underscores that even modest alcohol consumption can precipitate MALA in the presence of mild renal impairment.

## Introduction

Metformin is widely prescribed as the first-line therapy for type 2 diabetes mellitus owing to its cardiovascular benefits, safety profile, and low cost [1], and it is also recommended as the first-line agent in the latest American Diabetes Association guideline [2]. Nevertheless, metformin-associated lactic acidosis (MALA) remains a rare but potentially life-threatening adverse effect, with a reported mortality rate of 30-50% despite its low incidence [3]. Therefore, prompt recognition and appropriate management are essential. Alcohol consumption is recognized as a precipitating factor for MALA, and several cases associated with excessive alcohol intake have been described [4,5]. Although excessive alcohol intake is recognized as a precipitant, the threshold quantity capable of inducing MALA remains unclear. Recent pharmacovigilance data suggest that the overall frequency of MALA is extremely low, estimated at <10 cases per 100,000 patient-years [3]. This case is notable because it involves only 28 g of ethanol, representing one of the few documented instances of MALA triggered by modest alcohol consumption, highlighting a potential risk even with modest amounts. Here, we report the case of a man in his sixties, without a history of habitual heavy drinking, who developed MALA after ingesting a relatively modest amount of alcohol. This case highlights the potential risk of MALA induced by even a modest amount of alcohol consumption in patients receiving metformin.

## Case presentation

A 66-year-old man (height: 163 cm; weight: 60.3 kg) with a medical history of type 2 diabetes mellitus, hypertension, and angina pectoris presented to the emergency department with transient loss of consciousness. His medications included metformin 1,500 mg/day, empagliflozin 10 mg/day, linagliptin 5 mg/day, olmesartan 20 mg/day, azelnidipine 16 mg/day, carvedilol 10 mg/day, aspirin 100 mg/day, vonoprazan 10 mg/day, ezetimibe 10 mg/day, rosuvastatin 5 mg/day, and calcium polystyrene sulfonate 5.4 g/day. He reported occasional alcohol consumption (14 g/week) and denied smoking.

At 11:00 a.m. in June (ambient temperature 27.4 °C, humidity 60%), he visited a public bathing facility. After bathing for approximately 15 minutes, he consumed 700 mL of beer (estimated 28 g of ethanol). Approximately 100 minutes after alcohol consumption, he experienced a transient loss of consciousness while seated and was transported by ambulance. Upon arrival, he was alert and conversant without chest pain or palpitations, but was hypotensive.

Initial vital signs included a Glasgow Coma Scale score of E4V5M6, heart rate of 73 bpm (sinus rhythm), respiratory rate of 18 breaths per minute, blood pressure of 68/43 mmHg, oxygen saturation of 98% on room air, and temperature of 36.1°C. Physical examination was otherwise unremarkable. Electrocardiography and echocardiography were unremarkable. He had no history of recent surgery or gastrointestinal symptoms suggestive of malnutrition, and his dietary intake before the event was reported to be normal without any restriction or irregularities.

Laboratory findings revealed impaired renal function (blood urea nitrogen 27.0 mg/dL, creatinine 1.59 mg/dL, estimated glomerular filtration rate (eGFR) 35.1 mL/minute/1.73 m²), compared with three months earlier (creatinine 0.92 mg/dL, eGFR 64.1 mL/minute/1.73 m²), consistent with acute kidney injury. Electrolytes were within normal limits, serum ketone bodies (acetoacetate, β-hydroxybutyrate, and acetone) were negative, and blood alcohol concentration was 52.7 mg/dL. Arterial blood gas analysis showed a pH of 7.33, a bicarbonate level of 16.2 mmol/L, and a lactate level of 7.4 mmol/L, consistent with lactic acidosis. Other laboratory data are summarized in Table 1. Given the lactic acidosis together with his history of metformin use and alcohol intake, MALA was suspected.

**Table 1:** Laboratory tests on admission Reference ranges reflect institutional standards ^*^Abnormal values are bolded for quick recognition AG, anion gap; ALB, albumin; ALP, alkaline phosphatase; ALT, alanine transaminase; AST, aspartate aminotransferase; Bil, bilirubin; BUN, blood urea nitrogen; Ca, calcium; CK, creatine kinase; Cl, chloride; Cre, creatinine; CRP, C-reactive protein; eGFR, estimated glomerular filtration rate; γ-GTP, γ-glutamyl transferase; Glu, glucose; Hb, hemoglobin; Ht, hematocrit; HbA1c, hemoglobin A1c; HCO₃⁻, bicarbonate; K, potassium; LDH, lactate dehydrogenase; Mg, magnesium; Na, sodium; P, phosphorus; pCO₂, partial pressure of carbon dioxide; Plt, platelet; pO₂, partial pressure of oxygen; PT-INR, prothrombin time-international normalized ratio; UA, uric acid; WBC, white blood cell

Laboratory test (unit)	Admission laboratory results	Reference range
pH	7.33^*^	7.35-7.45
pCO_2_ (mmHg)	31.3^*^	35-48
pO_2_ (mmHg)	101.8	83-108
HCO_3_^-^ (mmol/L)	16.2^*^	21-28
AG (mmol/L)	20.1	10-20
Lactate (mmol/L)	7.4^*^	0.5-1.6
PT-INR	1.03	0.89-1.12
WBC (×100/µL)	91	33-86
Hb (g/dL)	11.2^*^	13.7-16.8
Ht (%)	37.2	35.1-44.4
Plt (×10,000/µL)	32.4	15.8-34.8
ALB (g/dL)	3.6^*^	4.1-5.1
BUN (mg/dL)	27^*^	8-20
Cre (mg/dL)	1.59^*^	0.65-1.07
eGFR (mL/minute/1.73 m^2^)	35.1^*^	＞60
UA (mg/dL)	5.7	3.7-7.8
Na (mmol/L)	138	138-145
K (mmol/L)	4.7	3.6-4.8
Cl (mmol/L)	103	101-108
Mg (mg/dL)	2	1.8-2.4
P (mg/dL)	3.7	2.7-4.6
Ca (mg/dL)	8.6^*^	8.8-10.1
AST (U/L)	14	13-30
ALT (U/L)	12	10-42
LDH (U/L)	187	124-222
ALP (U/L)	61	38-113
γ-GTP (U/L)	12^*^	13-64
Total Bil (mg/dL)	0.3^*^	0.4-1.5
CK (U/L)	55^*^	59-248
Glu (mg/dL)	98	73-109
HbA1c (%)	6.1	4.9-6.8
CRP (mg/dL)	0.015	0-0.14
Ketones	Negative	-
Alcohol (mg/dL）	52.7^*^	3-30

**Figure 1:**
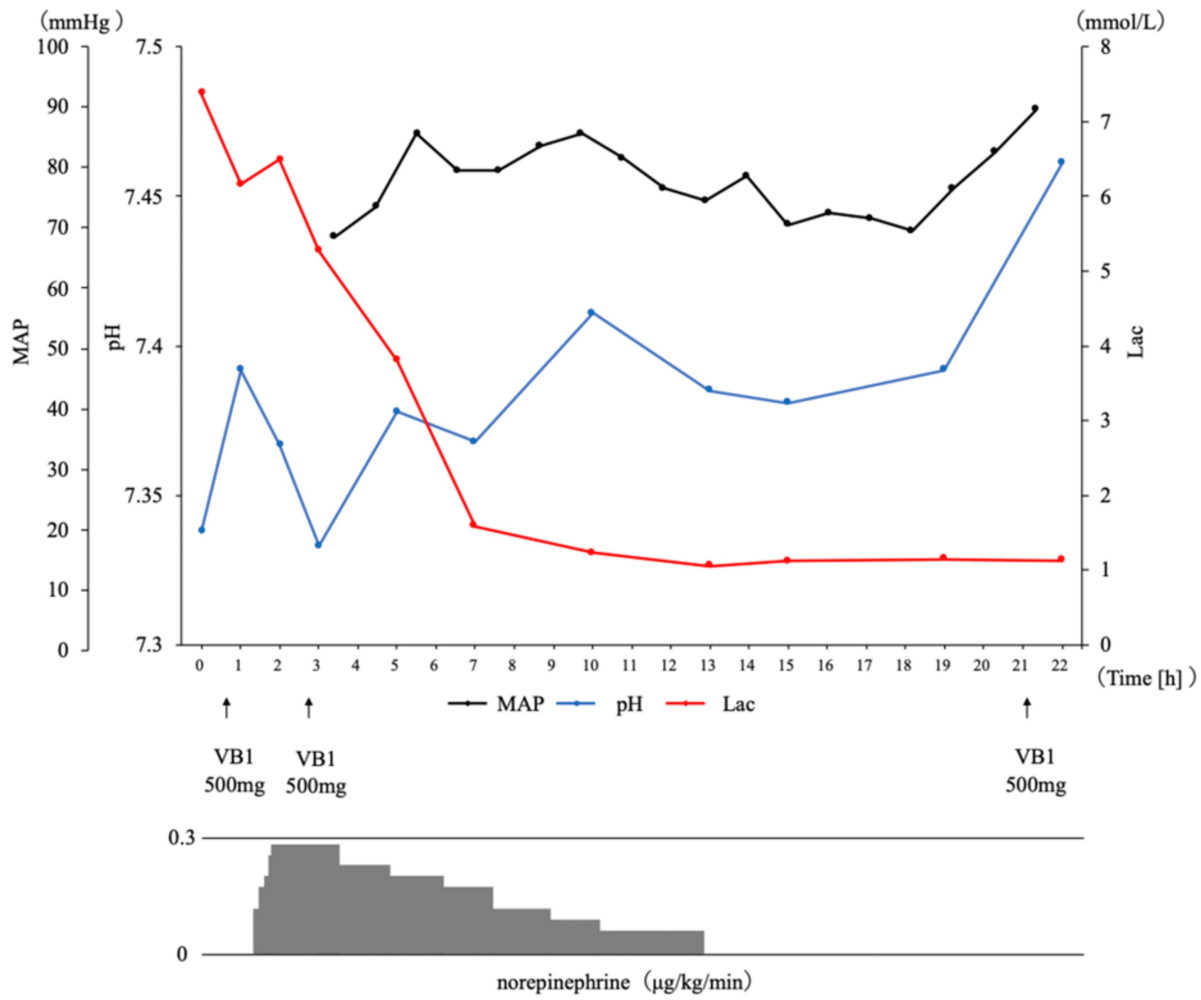
Clinical course after hospital admission Time course of mean arterial pressure, pH, and lactate up to 22 hours after admission, along with pharmacological interventions. The trajectory of norepinephrine infusion rates is also depicted Lac, lactate; MAP, mean arterial pressure; VB1, vitamin B1

Vitamin B1 (500 mg) was empirically administered to exclude lactic acidosis due to thiamine deficiency. Despite infusion of 1,500 mL of extracellular fluid, hypotension persisted, meeting criteria for intensive care unit (ICU) admission (persistent hypotension despite fluids), and norepinephrine at 0.28 μg/kg/minute was initiated. He was admitted to the ICU, considering the possibility of further deterioration. As blood pressure stabilized, norepinephrine was tapered and discontinued after approximately 12 hours. After 22 hours, laboratory results improved: lactate 1.1 mmol/L, pH 7.46, and bicarbonate 22.8 mmol/L (Figure 1).

Vitamin B1 levels obtained before supplementation were elevated (1,380.9 ng/mL), excluding deficiency; Vitamin B1 supplementation was discontinued after two days. On hospital day 2, he resumed oral intake and restarted his usual medications except metformin, and was discharged from the ICU. By day 3, creatinine had improved to 0.79 mg/dL and eGFR to 75.8 mL/minute/1.73 m², consistent with normalization of renal function. Blood pressure remained stable, symptoms had resolved, and metformin was permanently discontinued. He was discharged on hospital day 3. At outpatient follow-up, he remained stable, with normalization of renal function and no evidence of recurrence.

This case report did not fall under the scope of review by the Ethics Committee of Shonan Kamakura General Hospital. However, informed consent for publication was obtained from the patient, and careful attention was paid to protecting the patient’s privacy.

## Discussion

We report a case of MALA in a patient without a history of heavy alcohol consumption or dietary abnormalities, precipitated by the ingestion of a relatively modest amount of alcohol. While the quantity of alcohol in this case was lower than that reported in previous studies (Table 2), several additional factors likely contributed to the patient’s condition. These include moderate renal dysfunction (eGFR 35.1 mL/minute/1.73 m²), an excessive daily dose of metformin (1,500 mg/day), and transient dehydration following bathing. Taken together, these findings suggest that even modest amounts of alcohol may precipitate MALA in patients on metformin therapy, particularly when multiple predisposing factors are present.

**Table 2:** Clinical characteristics of patients with MALA and a history of alcohol consumption The columns indicate the clinical parameters compared, and the rows show the results from each referenced case report. Data were extracted from the present case and previously published reports [4-9] eGFR, estimated glomerular filtration rate; MALA, metformin-associated lactic acidosis

Parameter	Present case	Yamagishi et al. [6]	Suda et al. [5]	Fujita et al. [4]	Suzuki et al. [7]	Farouji et al. [8]	Rai et al. [9]
Age (years)	66	65	55	69	44	63	77
Sex	Male	Male	Male	Male	Female	Female	Male
Dosage of metformin (mg/day)	1,500	1,000	750	None noted	5,000	1,000	2,000
Alcohol consumption history	14 g/week	130-260 g/day	72 g/day	108 g/day	Occasional	28-42 g/day	Dependence
Alcohol intake (g/day)	28	260	135	216	90	None noted	None noted
Last intake of alcohol	Day of admission	Day of admission	Day of admission	Day of admission	Day of admission	None noted	None noted
Blood alcohol concentration at admission (mg/dL)	52.7	None noted	None noted	None noted	None noted	None noted	None noted
Serum creatinine concentration at admission (mg/dL)	1.59	1.4	2.7	1.4	0.94	1.5	7.39
eGFR at admission (mL/minute/1.73 m^2^)	35.1	40.5	28.7	None noted	None noted	None noted	6
pH	7.33	6.966	6.85	6.778	7.067	7.34	6.94
Lactate (mmol/L)	7.83	30	13.89	>20	13.2	13	13.1
Treatment	Infusion	Infusion	Dialysis	Dialysis	Dialysis	Dialysis	Dialysis
Outcome	Survival	Survival	Survival	Survival	Survival	Survival	Death
Length of hospital stay (days)	3	17	15	23	1 month	None noted	14
Outcome summary	6/7 survived (mortality rate 14%)						

Lactic acidosis is defined as blood lactate >5 mmol/L, pH <7.35, and bicarbonate <20 mmol/L [10]. Its common causes include cardiogenic, hypovolemic, or septic shock; tissue hypoxia due to carbon monoxide poisoning or severe anemia; and increased anaerobic metabolism during seizures. Impaired lactate metabolism may also occur in hepatic or renal dysfunction, malignancy, thiamine deficiency, or drug-induced etiologies, such as alcohol intoxication, propofol, linezolid, or antiretroviral therapy [10].

In this case, the patient met the diagnostic criteria for lactic acidosis. He presented with persistent hypotension and lactic acidosis after bathing and alcohol intake, requiring vasopressor support. We initially considered other causes of shock, including cardiogenic, septic, and hypovolemic shock, but the clinical findings were not compatible with these etiologies as the primary cause. Transient hypotension due to dehydration typically responds to fluid resuscitation alone; however, the prolonged lactic acidosis requiring vasopressors in this patient was most consistent with metformin use. In addition, there was no evidence of malignancy, malnutrition, thiamine deficiency, or other drug-induced etiologies causing lactic acidosis. Given that he was receiving metformin and other causes were excluded, we finally diagnosed the condition as MALA [3].

Reported risk factors for MALA include renal dysfunction, dehydration, acute illness, excessive alcohol consumption, cardiovascular or pulmonary dysfunction, perioperative state, hepatic impairment, and advanced age [3,11]. Importantly, MALA may occur even in relatively young patients or in those on low-dose metformin when risk factors are present.

In this case, dehydration likely developed after alcohol consumption following bathing. On admission, the patient’s eGFR was 35.1 mL/minute/1.73 m², indicating moderate renal dysfunction. Both dehydration and acute kidney injury likely contributed to the onset of MALA. At this level of renal function (eGFR 30-45 mL/minute/1.73 m²), the recommended maximum daily dose of metformin is 750-1,000 mg [12,13]. Therefore, the prescribed dose of 1,500 mg/day exceeded the recommended limit and may have led to transiently elevated serum metformin concentrations, thereby increasing the risk of MALA [14]. 

Although the alcohol intake in the case was modest (28 g), MALA developed shortly thereafter, suggesting a contributory role. During ethanol metabolism, nicotinamide adenine dinucleotide (NAD⁺) is reduced to its nicotinamide adenine dinucleotide (NADH; reduced form), leading to an increased NADH/NAD⁺ ratio. This shift promotes the conversion of pyruvate to lactate, inhibits pyruvate dehydrogenase activity, and suppresses hepatic gluconeogenesis, thereby impairing lactate clearance [15]. Metformin exerts similar redox-dependent effects by inhibiting mitochondrial complex I and suppressing hepatic gluconeogenesis [16]. Consequently, ethanol- and metformin-induced NADH accumulation synergistically inhibits hepatic lactate clearance and promotes lactate accumulation, predisposing the patient to MALA during acute alcohol intoxication. Previous reports have linked alcohol-induced MALA with ethanol intake exceeding 100 g in a single episode [3,4,6]. In contrast, our case developed MALA after ingesting approximately 28 g of ethanol (equivalent to about 700 mL of beer) during an ordinary daily situation (drinking after bathing), highlighting the potential for MALA even after modest alcohol consumption.

The treatment of MALA is mainly supportive, with hemodialysis reserved for severe cases to enhance metformin clearance [3,4,7-9]. In this case, metformin was discontinued, and circulatory support with intravenous fluids and norepinephrine led to rapid improvement in hemodynamics, lactate levels, and metabolic acidosis. He was discharged three days after admission. Compared with previously reported cases of alcohol-induced MALA requiring hemodialysis, this case was relatively mild [3,4,7-9]. Nevertheless, the episode of syncope and hypotension on presentation underscores that, without timely intervention, the patient might have developed life-threatening complications such as multi-organ failure.

Among the seven reported cases of alcohol-associated MALA (Table 2), six patients survived and one patient died, corresponding to a mortality rate of approximately 14%. These findings highlight that alcohol-associated MALA can be severe and potentially fatal, even when triggered by modest alcohol intake. Preventive measures should include appropriate dose adjustment of metformin according to renal function, avoidance of alcohol intake during dehydration or illness, and patient education regarding the synergistic risk of alcohol and metformin on lactate metabolism.

## Conclusions

We report a case of MALA in a diabetic patient without a history of heavy alcohol use, who developed the condition after modest alcohol ingestion. This case suggests that even modest alcohol intake may precipitate MALA in patients on metformin therapy. Clinicians should consider adjusting metformin doses or temporarily withholding therapy during episodes of dehydration or alcohol consumption. In addition, careful monitoring of renal function and patient education regarding the combined risks of metformin and alcohol are recommended to prevent similar events.
